# Higher levels of multimorbidity are associated with increased risk of readmission for older people during post-acute transitional care

**DOI:** 10.1007/s41999-023-00770-5

**Published:** 2023-04-03

**Authors:** Ornagh Griffin, Tracy Li, Alexander Beveridge, Danielle Ní Chróinín

**Affiliations:** 1 Department of Geriatric Medicine, St Vincent’s Hospital, Sydney, NSW Australia; 2 St. Vincent’s Clinical School, UNSW Sydney, Sydney, NSW Australia; 3 Department of Geriatric Medicine, Liverpool Hospital, Corner of Elizabeth and Goulburn St, Liverpool, NSW Australia; 4 South Western Sydney Clinical School, UNSW Sydney, Sydney, NSW Australia

**Keywords:** Multimorbidity, Transitional care, Readmission, Aged care

## Abstract

**Purpose:**

Older patients are at high risk for poor outcomes after an acute hospital admission. The Transitional Aged Care Programme (TACP) was established by the Australian government to provide a short-term care service aiming to optimise functional independence following hospital discharge. We aim to investigate the association between multimorbidity and readmission amongst patients on TACP.

**Methods:**

Retrospective cohort study of all TACP patients over 12 months. Multimorbidity was defined using the Charlson Comorbidity Index (CCI), and prolonged TACP (pTACP) as TACP ≥ 8 weeks.

**Results:**

Amongst 227 TACP patients, the mean age was 83.3 ± 8.0 years, and 142 (62.6%) were females. The median length-of-stay on TACP was 8 weeks (IQR 5–9.67), and median CCI 7 (IQR 6–8). 21.6% were readmitted to hospital. Amongst the remainder, 26.9% remained at home independently, 49.3% remained home with supports; < 1% were transferred to a residential facility (0.9%) or died (0.9%). Hospital readmission rates increased with multimorbidity (OR 1.37 per unit increase in CCI, 95% CI 1.18–1.60, *p* < 0.001). On multivariable logistic regression analysis, including polypharmacy, CCI, and living alone, CCI remained independently associated with 30-day readmission (aOR 1.43, 95% CI 1.22–1.68, *p* < 0.001).

**Conclusions:**

CCI is independently associated with a 30-day hospital readmission in TACP cohort. Identifying vulnerability to readmission, such as multimorbidity, may allow future exploration of targeted interventions.

## Key summary points

**Aim:**

To investigate the association between multimorbidity and readmission amongst patients on Transitional Aged Care Program (TACP).

**Findings:**

Hospital readmission rates increased with multimorbidity and the Charlson Comorbidity Index (CCI) is independently associated with a 30-day hospital readmission in TACP cohort.

**Message:**

Identifying vulnerability to readmission, such as multimorbidity may allow future exploration of targeted interventions to optimise transitional care and individualise patient care to improve functional independence and prevent premature Residential Aged Care Facilities (RACF) admissions in older people.

## Introduction

During hospitalisation, older people are at risk of significant functional decline, which may impair their future independence and quality of life [1], and a recent Australian paper noted that in-hospital functional decline occurred in two thirds of patients [2]. Loss of independence in these activities is strongly associated with institutionalization, caregiver burden, higher resource use, and death [1, 3]. High readmission rates may be a marker of poor quality of care [4] as well as an economic burden [5]. With increasing numbers of older people admitted to hospital, there comes too the need to facilitate efficient discharge planning, aiming to prevent long hospital admissions, premature permanent Residential Aged Care Facilities (RACF) admissions and reduce hospital readmissions.

The prevalence of multimorbidity in older populations is high; amongst Australians aged 65 years or older, 60% have two or more chronic conditions [6]; this is not dissimilar to other regions [7]. Multimorbidity is associated with more complex health needs, prolonged acute hospital admissions and increased frequency of readmissions [8, 9]. It may thus be intuitive that patients with multimorbidity might benefit from coordinated and continuing care post-discharge, with a focus on functional restoration.

The Transitional Aged Care Programme (TACP) was established by the Australian government in 2005 to provide short-term support and post-acute rehabilitation for people aged 65 years or older at discharge from acute/subacute care to home [10]. Although there is no established transitional care programme in Europe, studies are currently being conducted to assess the outcomes of transitional care programmes [11] and a recent multicentre randomised trial conducted in the Netherlands observed that acutely hospitalised older patients  > 65 years old who received a comprehensive geriatric assessment (CGA) combined with transitional care had lower 1-month and 6-month mortality rates compared to those who receive CGA alone [12]. Transitional care is short-term care whose aim is to optimise an older person’s function and independence following hospital discharge. TACP provides a community based, case-managed, goal-orientated and time-limited, flexible package of services including physical therapy and personal or home care services provided by a multidisciplinary team. TACP is usually delivered at a recipient’s home and is initially approved for up to 12 weeks, with a 6-week extension possible. According to the Australian Institute of Health and Welfare, the average duration of transitional care is 60 days [10].

Transitional care between hospital and home have been shown to be effective in improving patient’s functional status and allowing older patients to remain home after an acute hospital admission, in Australia and elsewhere [12–14]. Cations et al. [15] found that for all older Australians accessing TACP between 2007 and 2015, functional independence improved from entry to discharge for almost 4 in 10 people, with 50% of TACP users were able to remain home after 6 months. Entry to permanent care was more common with older age, higher levels of frailty and those with dementia. The effectiveness of TACP was further highlighted in a study of 369 patients who underwent residential TACP, almost 70% were able to return home [16].

The Charlson Comorbidity Index (CCI) is the most frequently used tool to measure co-existing disease and has been validated in the older population as a predictor of mortality [17, 18]. Higher CCI scores are associated with increased readmission rate following discharge from hospital as evident in patients following orthopaedic surgery [19] and has been associated with increasing dependence and frailty [20]. Composite measures including the CCI have been investigated for increased ability to predict the risk of death or unplanned readmissions within 30 days after hospital discharge [21]. Other patient factors may also be associated with increased likelihood of readmission within a 30 day period, across various groups, and most not particular to patients availing of TACP. These include social isolation, polypharmacy (defined as ≥ 5 medications), and a variety of specific medical conditions, such as osteoarthritis and dementia [22–28]. Frailty itself may also be associated with increased hospital readmission within 30 days [29, 30]. Many of these factors are indeed associated with a plethora of adverse outcomes beyond readmission, including emergency admissions, hospital length-of-stay, and mortality [31]. Looking specifically at TACP cohorts, a New Zealand group identified that poorer functional status and comorbidity burden were associated with readmission where readmission rates were high at 42% [32]. The intervention structure may also affect outcome. Nonetheless, a review of the literature highlights several evidence gaps in terms of outcomes in patients receiving transitional care [15].

In this context, we sought to identify whether multimorbidity, as determined by CCI, was associated with an increased risk of 30 day hospital readmission while in receipt of transition care at home, and whether associated with duration of needing TACP.

## Methodology

### Inclusion criteria and admission to TACP

We conducted a retrospective observational study of all patients discharged from an in-patient setting to a single-centre Transitional Aged Care Programme (TACP), in Sydney, for the year July 2013–June 2014 inclusive. Patients were admitted to this programme from a tertiary university teaching hospital in New South Wales (NSW), Australia, and to a lesser extent from the local rehabilitation (subacute) hospital and other nearby hospitals (public/private). Patients were referred by their in-patient team once medically well and stable for transfer to the community but identified as requiring ongoing involvement of at least 2 allied health disciplines to optimise their community reengagement. Patients were identified as potentially suitable for TACP by the treating multidisciplinary team, in consultation with a geriatrician, and referred to the local Aged Care Assessment Team (ACAT). ACAT uses standardised eligibility criteria to determine whether a person is suitable for TACP, including that they are currently an in-patient, able to enter the care programme within 24–48 h of discharge, and who are deemed likely to benefit from goal-oriented, time-limited care and support in a non-hospital environment to “complete their restorative process, optimise their functional capacity, and finalise and access their longer term care arrangements” [10].

Length-of-stay on TACP was determined by the TACP team, based on achievement of pre-defined individualised rehabilitation goals; when these were reached, patients were discharged from the programme.

### Data sources and variables

Patient electronic notes and TACP databases were reviewed to identify patient demographics, multimorbidity, functional independence, medications, length-of-stay on TACP, and readmission (to an acute hospital) within 30 days. Primary diagnoses were determined by the treating medical team. The primary outcome measure was 30-day readmission (to an acute hospital), with length-of-stay on TACP programme and prolonged TACP admission as secondary outcome measures.

Community discharge supports at time of exit from the TACP were ordered according to the level of support offered, and analysed as an ordinal variable, from independent living (no additional formal supports), those at home with formal supports (eg: government funded homecare packages or private carers) and to those requiring residential care.

### Definition of multimorbidity

Functional independence was measured using the Barthel index (score out of 100) which was performed by the TACP therapist. The CCI was used to measure burden of disease. Data from each patient’s chart was entered into the CCI table to formulate a numerical result. We defined high CCI as CCI in the highest quartile (CCI > 9).

Musculoskeletal disorder included any patients with osteoporosis, osteoarthritis or gout.

Gait disorder included any patient with a history of falls, or requiring a walking aid as documented by physiotherapist of the treating medical team. This included patients whose primary diagnosis (eg: fall with a fractured pelvis) resulted in the need of a walking aid at discharge. Cognitive impairment was documented if a score of 26 or below was recorded in medical notes from formal cognitive testing using the Mini Mental State Examination (MMSE) or similar screening tool. Mental health disorders included the documentation of depression, or other psychiatric illnesses. Social supports were identified on the initial assessment by TACP case manager. Polypharmacy was defined as receiving > 5 medications on admission to TACP.

Gait disorder referenced any TACP patient documented to have had a fall or using a walking aide as documented on discharge. 'Gait disorder' was diagnosed by the treating TACP physiotherapist, and based on history and examination, including the use of standardised objective measures (Timed up and Go, 10 Metre walk test, 6 Minute Walk Test and included gait disorders of varying aetiology and phenotype [33].

### Outcome measures

The primary outcome measure was readmission to the acute hospital. Only index admissions to TACP were included. Secondary outcome measures included prolonged TACP (pTACP), defined as  ≥ 8 weeks on the TACP programme, and level of support needed at discharge from TACP. We also recorded patient death.

Formal services were defined as paid/funded support social/health services, beyond ‘informal’ (unpaid) care by family/friends. Gait disorder was captured as present where diagnosed by the treating TACP team (who specifically collected this information) and cognitive impairment included a medical diagnosis of dementia, mild cognitive impairment or cognitive impairment not otherwise specified.

TACP covers all of Australia, but is broken up into local service providers, based on geographical area. Since its inception in 2005/6 to 2017/18, nationally, over 187,000 care recipients had received TACP, with 25,113 transitional aged care recipients in 2017/18 [34]. The TACP service within St Vincent’s Health Network Sydney is located in the geographical boundaries of the South Eastern Sydney and Western Sydney Local Health Districts. This TACP team services a catchment area which was home to > 38,000 people aged ≥ 65 at that time.

### Statistical analysis

Association between outcomes (dichotomised) and variables of interest were analysed using logistic regression analysis. Independent variables were considered for inclusion in the models if biologically plausible and/or supported by the available literature and available within our data sources. Variables which were associated with outcome on univariate analysis were included in a multivariable model. Variables which were components of the CCI were excluded from models including the CCI score, to avoid collinearity [17, 18]. For multivariable analysis, an a priori strategy was used, where factors strongly associated with outcome in the existing literature were included in the multivariable model, even where they did not reach statistical significance in our own univariate analysis, unless they were collinear (correlation coefficient ≥ 0.5) with other included variables. Characteristics shown in Table 2 were included in univariate analyses (including age, sex, referral source, morbidities detailed, polypharmacy, living situation, social supports). While select morbidities were analysed (mental health disorder, musculoskeletal disorder, gait disorder, cognitive impairment), variables included in the CCI were not otherwise extracted for individual analysis. To determine whether the CCI may be helpful in predicting 30-day readmission, we employed the Receiver Operator Characteristic (ROC) curve test.

Statistical analyses were performed using Stata v13.0.

### Ethical approval

This study was approved as a Low to Negligible Risk project by St Vincent’s Human Research Ethics Committee (SCH14/029), and the need for individual patient consent was waived.

## Results

Overall, 227 patients received TACP upon discharge from hospital in the year July 2013–June 2014, and were eligible for inclusion. No variables for investigation were missing, and no patients were lost to follow-up. Overall, the mean age was 83.3 ± 8.0 years, and 142 (62.6%) were female. A third (32.2%) had documented formal services prior to admission. Principal diagnosis for initial hospital admission were categorised into systems groups as shown in Table 1. ‘Musculoskeletal and mobility problems’ comprised the commonest diagnostic group, most often consisting of a fall. In total, 80/227 (45.2% of whole group) patients presented with fall, and over half of these (49/80) were admitted with fall and associated fracture. The commonest cardiorespiratory problem was lower respiratory tract infection/pneumonia (N = 18) followed by CCF (N = 5). The majority of patients were admitted to TACP from the acute public hospital, with the distribution of ward sources shown in Table 2, with a further 20% from the local rehabilitation hospital, and a smaller proportion from private hospitals.

**Table 1:** Principal diagnostic groups for initial hospital admission

Principal diagnosis	Frequency,* N* (%)
Musculoskeletal and mobility problems	124 (54.6)
(a) Fall without fracture	31 (13.7)
(b) Fall with fracture	49 (21.6)
(c) Musculoskeletal	44 (19.4)
Cardiothoracic	39 (17.2)
Delirium	9 (4.0)
Cerebrovascular accident	13 (5.7)
Gastrointestinal conditions	12 (5.3)
Cellulitis	5 (2.2)
Sepsis	5 (2.2)
Urology	8 (3.5)
Other	12 (5.3)

**Table 2:** Baseline patient characteristics

Characteristics	Overall
Demographics	
Overall, n (%)	227 (100)
Mean age (SD)	83.3 (8.0)
Female, n (%)	142 (62.6)
Referral specialities	
General geriatric ward, n (%)	36 (15.9)
Geriatric acute medical unit, n (%)	24 (10.6)
Other wards, n (%)	41 (18.0)
Local private rehab hospital, n (%)	39 (17.2)
Local public rehabilitation hospital, n (%)	52 (22.9)
Other hospitals within metropolitan area, n (%)	35 (15.4)
Polypharmacy, n (%)	207 (92)
Multimorbidity	
Mental health disorder, n (%)	60 (26.4)
Musculoskeletal disorder, n (%)	162 (71.4)
Gait disorder, n (%)	208 (91.6)
Cognitive impairment, n (%)	127 (56.0)
CCI, median (IQR)	7 (6–8)
Living situation	
Living with someone, total n (%)	84 (37.2)
(a) Without formal support services, n (%)	57 (25.2)
(b) With formal support services, n (%)	27 (12.0)
Living alone with supportive family and friends, total n (%)	72 (31.9)
(a) Without formal support services, n (%)	39 (17.3)
(b) With formal support services, n (%)	33 (14.6)
Lived alone, total n (%)	70 (31.0)
(a) Without formal support services, n (%)	57 (25.2)
(b) With formal support services, n (%)	13 (5.8)
Outcomes	
Readmission to hospital, n (%)	50 (22.0)
Stayed at home with formal services, n (%)	112 (49.3)
Stayed at home with no services, n (%)	61 (26.9)
Residential aged care facility, n (%)	2 (0.88)
Death, n (%)	2 (0.88)

Multimorbidity are shown in Table 2. Evidence of a gait disorder was very common, present in 9/10 patients, and over half had cognitive impairment. Polypharmacy was evident in 92% (207/227) of patients. Mean number of medications on admission to the TACP was 9.8 (SD 4.2).

At admission to TACP, over a third of the cohort lived with someone, and less than a third of the cohort lived alone without major support from family/friends (Table 2). The admission to TACP Barthel Index mean was 80.1 (SD 14.5) and mean Barthel Index at discharge from TACP was 92.6 (SD9.5).

Rates of multimorbidity were high. The median CCI for all patients was 7 (IQR 6–8) and 10% of patients had a CCI ≥ 11. Highest quartile of CCI was identified as CCI > 9.

### Patient outcomes

Median length-of-stay for all patients on TACP was 8 weeks (IQR 5–9.67). Overall, 37.9% of the total participants required prolonged TACP (≥ 8 weeks).

Just over 1 in 5 (22.0%, 50/227) of the TACP participants were readmitted within 30 days of discharge. These patients were automatically discharged from TACP at the time of hospital readmission.

Amongst those who were not readmitted, outcomes at the end of TACP are shown in Table 2; nearly half (49.3%) of patients remained at home with formal services, while very few died while on TACP or died (< 1% for each).

### Associations with and prediction of readmission to an acute hospital whilst on TACP

**Fig. 1:**
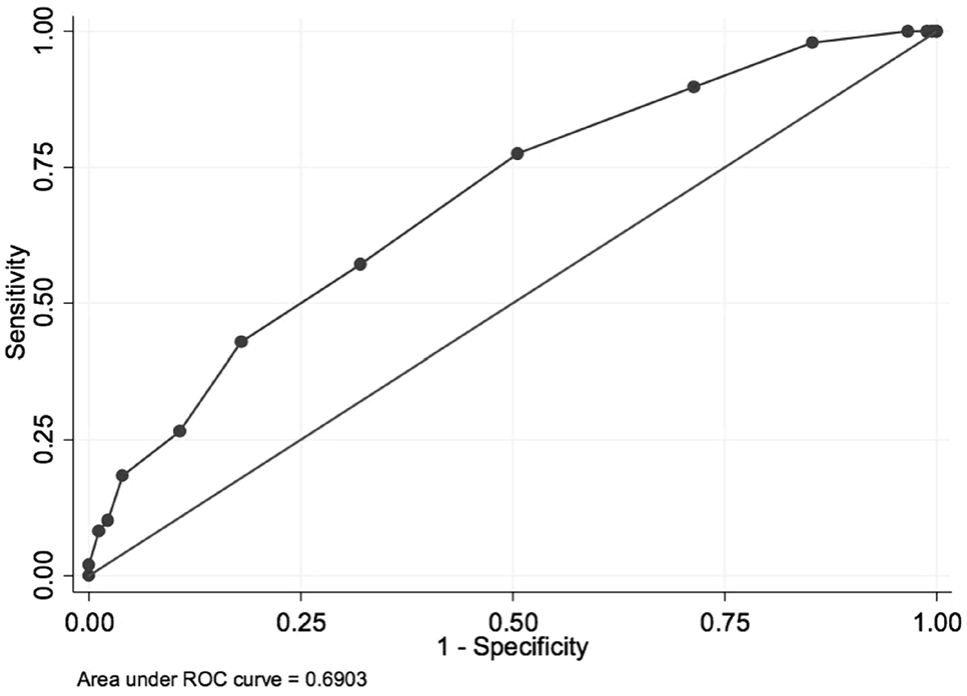
Charlson Comorbidity Index as a predictor of readmission rate; AUC 0.69. *AUC* area under the curve

On unadjusted logistic regression analysis, each point increase in CCI was associated with a 37% increase in the odds of requiring readmission to an acute hospital (unadjusted OR 1.37 per unit increase, 95% CI 1.18–1.60,* p* < 0.001). Likewise, when analysed as a binary variable, the highest quartile of CCI was associated with readmission within 30 days (unadjusted OR 3.42 with CI 1.72–6.77,* p *< 0.001). CCI was a moderately strong predictor of readmission, with an area under the ROC curve of 0.69 (Fig. 1).

On univariate analysis, we did not observe any association between readmission and any of the other investigated variables. However, based on previous published evidence indicating a strong association between early hospital readmission and both polypharmacy and social supports, and as per our a priori statistical strategy, we included these in our final multivariable model. On multivariable logistic regression analysis, adjusting for both polypharmacy and social supports, CCI retained an independent association with 30-day readmission (aOR 1.43, 95% CI 1.22–1.68,* p* < 0.001) (Table 3a). Likewise, when analysing highest quartile of CCI, only high CCI (aOR 3.9, CI 1.9–8.01,* p* < 0.001) was independently associated with likelihood of 30-day readmission (Table 3).

**Table 3:** Multivariable logistic regression analysis of likelihood of 30-day readmission, (a) with CCI as continuous variable, (b) including highest quartile of CCI

	Odds Ratio	95% CI	*p* value
(a)			
CCI (per unit increase)	1.43	1.22–1.68	0.00
Lives alone	1.54	0.99–2.39	0.05
Polypharmacy	1.11	0.29–4.28	0.88
(b)			
Highest quartile CCI	3.90	1.90–8.01	0.00
Lives alone	1.37	0.90–2.08	0.14
Polypharmacy	1.12	0.30–4.17	0.86

### Secondary analyses: associations with prolonged TACP, level of support and death

CCI score was inversely associated with prolonged TACP course (OR 0.87 per unit increase, 95% CI 0.77–0.99,* p *= 0.04). Those requiring more support at time of discharge from TACP had increased likelihood of pTACP (OR per increased level of support 1.72, CI 1.19–2.489,* p* = 0.004). While polypharmacy was not associated with pTACP on our univariate analysis, it was included in the multivariable model as per our a priori research strategy. On multivariable logistic regression analysis, in a model including CCI, polypharmacy, and social supports, CCI was no longer inversely associated with pTACP (OR 0.93,* p* = 0.31). Findings were unchanged when polypharmacy was excluded from the model.

On a sensitivity analysis, when patients who required readmission within 30 days were excluded, the inverse association between CCI and prolonged TACP admission was no longer observed.

CCI was not associated with level of support required on discharge from TACP. Numbers of decedents were too small (*N* = 2) to allow for meaningful analysis of any association between CCI and death.

## Discussion

In this retrospective review of 227 consecutive patients admitted to our TACP programme, multimorbidity, as represented by the CCI, was associated with 30-day readmission to an acute hospital. This was the case when CCI was analysed either as a continuous variable or dichotomised (highest quartile versus other), and an independent association between CCI and risk of readmission remained on adjusted analysis.

While evidence suggests that multidisciplinary aged care team interventions may reduce readmission rates in older patients discharged from the emergency department or in-patient wards [35, 36], we note that in the specific group of older patients receiving transitional aged care on hospital discharge, CCI may have moderate utility in identifying those at higher risk of rehospitalisation. Our evidence, drawn retrospectively from a single TACP area, indicates that the CCI may have some utility in predicting readmission amongst those receiving multidisciplinary transitional care. The CCI is easily calculated using data routinely collected in clinical practise. The ROC curve we observed, with AUC 0.69, suggests that the CCI is moderately predictive of readmission, but is not without flaws for use as a predictive tool, and our data must be interpreted with caution. Existing algorithms have shown similar prediction values at best. The HOPE index [37], for example, developed and validated in over 3000 patients over a 24 month period, resulted in an AUC of 0.69 for mortality on ROC analysis, and AUC of 0.62 for rehospitalisation. Given data suggesting that other variables such as frailty [29], and dementia [23] are associated with readmission, a prospective study, exploring the potential increases in sensitivity and specificity if other factors are combined with CCI may be helpful in determining those at highest risk of hospital readmission.

While our initial analysis indicated that CCI was associated with lower odds of needing a longer TACP package, this signal was obviated on multivariable analysis, and when those who were readmitted were excluded. We hypothesise that this latter finding is because those with high levels of multimorbidity are at higher risk of readmission, thus cutting their TACP duration short.

Strengths of our study are the inclusion of a large cohort of otherwise unselected older persons who were admitted to our TACP programme over an entire year. We included consecutive patients, and no patients were lost to follow-up. In an unselected group of emergency department patients aged ≥ 75, a Sydney group noted 30-day readmission rates of only 17% in the group randomised to comprehensive geriatric assessment and 28-day multidisciplinary intervention [35]. Our TACP patients tend to represent the more vulnerable end of the community-dwelling aged population, and rates of multimorbidity were high; they were also transferring to TACP following a period of hospitalisation. A recent New Zealand study with similar numbers reported 3-month readmission rates of 42% following admission to a Community Rehabilitation, Enablement and Support (CREST) programme, similar to our TACP [32]. We did not identify any larger studies investigating factors associated with rehospitalisation in populations receiving multidisciplinary transitional aged care. Our data collection was comprehensive, with no missing relevant variables. Diagnoses and baseline multimorbidity were determined by the treating medical team and additional assessments and documentation of cognitive impairment, Barthel’s Indices, formal services, living situations, were all performed by accredited members of the TACP staff.

We acknowledge limitations of this study. This was a retrospective study for transition of care from a single-centre urban setting, where patients were discharged from either an acute hospital or in-patient rehabilitation unit to home. There may be under-reporting of some variables e.g. mental health, which were determined on history-taking and thus subject to reporting or recall biases. The data are now a few years old, and it is possible that characteristics of patients may have changed in this time period [34]. However, it retains relevance as high readmission rates are still noted in older and frail patients from programmes such as TACP through Australia and further afield [38]. Identification of vulnerabilities pre-discharged may potentially prevent further readmissions. As outlined in our results, this was a patient cohort entering a goal-orientated programme, therefore our findings in relation to readmission rates may not be necessarily generalizable to a non-TACP/rehabilitation cohort. We did not collect data identifying the causal factors prompting readmission, nor the outcomes of those patients who were readmitted such as death or transfer to permanent residential care. Heppenstall et al. found that half of readmissions amongst their CREST cohort (N = 224) were due to new acute medical problems, and a quarter due to exacerbation of chronic conditions; 86% of those who were readmitted were able to return home following hospitalisation [32]. Factors such as nutritional status [39, 40] and frailty indices [29] may be associated with adverse patient outcomes, and would have been good to include as stand-alone variables, but we did not have these data for our cohort. Finally, our mortality rates were low, precluding in-depth exploration of factors associated with death, but this is unlikely to be a frequent outcome in TACP cohorts.

It may be intuitive that higher multimorbidity might increase risk of readmission. However, to date, with the exception of that by Heppenstall et al. [32], there has been limited literature investigating the association between multimorbidity and readmission rates in patients admitted to TACP or similar community transition rehabilitation programmes. Prospective investigation of tools such as CCI, and potential refinement of predictive ability with addition of other easily accessible clinical data, may improve our ability to identify patients at highest risk of readmission, and determine whether targeted interventions can reduce risk and improve long term outcomes. This may improve the selection of patients, improve communication regarding likely outcomes with patients and their carers, and potentially allow us to better individualise care and target resources to groups most likely to benefit. This may be complimented by standardised data collection, and inclusion of readily available clinical and outcome variables in such databases.

In a moderately large group of consecutive patients admitted to TACP, we found that over 1 in 5 patients was readmitted to hospital within 30 days, and this was commoner in patients with higher levels of multimorbidity. Further study of readmission presentations may help stratify patients who may benefit further care planning prior to discharge and allow exploration of targeted interventions to reduce risk. This is important as poorly managed transitional care in older patients can lead to negative health outcomes and high costs for society. At present, findings from our current study are hypothesis-generating, but highlight vulnerability to readmission in older patients with high levels of multimorbidity receiving TACP.
